# Associations between Waist Circumference and Sex Steroid Hormones in US Adult Men: Cross-Sectional Findings from the NHANES 2013–2016

**DOI:** 10.1155/2024/4306797

**Published:** 2024-08-26

**Authors:** Zhisheng Zhu, Xingong Lin, Chaoyang Wang, Shize Zhu, Xianying Zhou

**Affiliations:** Plastic Surgery Second Affiliated Hospital of Fujian Medical University, Quanzhou 362000, China

## Abstract

**Background:**

Obesity is recognized as a major public health issue worldwide, characterized by a growing prevalence among adult males. Several studies have identified an association between obesity and sex steroid hormone levels but only a few have considered the relationship between waist circumference (WC) and sex hormone levels in adult males. This study therefore aimed to evaluate the relationships between waist circumference (WC) and various sex steroid hormone levels in adult males in the United States.

**Methods:**

This study analyzed data from 3,359 adult males aged 20 years and above, who participated in the National Health and Nutrition Examination Survey (NHANES) from 2013–2016 in the United States. We collected demographic data, including WC, and serum levels of testosterone, estradiol, SHBG, FAI, and T/E_2_ ratio. We adjusted the variables using multiple linear regression models with R 4.2.2 and EmpowerStats.

**Results:**

After adjusting for confounders, WC was found to be negatively associated with testosterone (*β* = −0.117, *P* < 0.001) but positively correlated with estradiol (*β* = 0.002, *P*=0.002), especially beyond a WC of 104.5 cm (*β* = 0.004, *P* < 0.001). Underweight individuals showed a contrasting positive correlation between WC and testosterone (*β* = 0.351, *P*=0.016). WC was inversely related to SHBG, particularly when WC was ≤99.1 cm (*β* = −0.036, *P* < 0.001). The FAI initially increased and then decreased with WC, peaking at 98.6 cm. The T/E_2_ ratio negatively correlated with WC (*β* = −0.074, *P* < 0.001). These relationships varied among subgroups but remained unaffected by age or physical activity time.

**Conclusions:**

Waist circumference is inversely correlated with testosterone, SHBG, and T/E_2_ ratio but positively correlated with estradiol, except for a positive correlation with testosterone in underweight males. Waist circumference serves as a crucial anthropometric measurement indicator for predicting sex steroid hormone levels in adult males.

## 1. Introduction

Since 1980, the prevalence of obesity has doubled in over 70 countries worldwide and continues to rise globally [1]. The increase in obesity poses a significant global health challenge, substantially affecting disease burden and incidence rates [2]. Waist circumference (WC), a simple and clinically practical method, is used to assess abdominal fat accumulation [3]. Compared to waist-to-hip ratio, weight, and body mass index (BMI), studies have confirmed that WC can more accurately predict a range of health issues, including coronary artery disease, type 2 diabetes, metabolic syndrome, hypertension, and abnormal cholesterol levels [4]. In addition, research by Yassin et al. has demonstrated that in men with hypogonadism, WC provides a more precise prediction of health-related symptoms than either weight or BMI [4]. Given these findings, promoting the use of WC measurement as a reliable and effective tool for assessing abdominal fat becomes particularly crucial in addressing the health challenges posed by global obesity.

Testosterone and estradiol play crucial roles in human growth and health [5]. They primarily regulate reproductive functions by influencing spermatogenesis in males and follicular development in females [6]. Imbalances in testosterone or estradiol levels may be associated with an increased risk of cardiovascular diseases, diabetes, and cancers [7]. Sex hormone-binding globulin (SHBG), a protein secreted by the liver, binds testosterone and estradiol in the blood with high affinity, regulating their bioactivities [8]. Hypogonadism, a condition closely related to obesity, affects approximately one-third of obese men and can exacerbate gonadal dysfunctions [9]. This condition is characterized by reduced secretion of testicular androgens, which not only decreases fertility and sexual function but may also lead to dysregulated fat metabolism, increased fat deposition, and elevated body mass index, ultimately promoting obesity [10]. Furthermore, obesity can induce a decrease in SHBG levels through mechanisms such as insulin resistance and proinflammatory cytokines. A reduction in SHBG levels leads to increased levels of free testosterone, which may enhance the aromatization of testosterone to estrogen in the adipose tissue. Elevated estrogen levels could, through a negative feedback loop, impact the hypothalamic-pituitary-gonadal axis, reducing the production of gonadotropin-releasing hormone, thereby lowering testosterone production and increasing fat deposition [9]. This cascade further exacerbates the progression of obesity and increases WC in adult men.

In the recent study, Wang et al. investigated a cohort comprising 4,031 children and adolescents, aged 6 to 19 years, examining the correlation between WC and levels of sex hormones, including total testosterone, estradiol, sex hormone-binding globulin (SHBG), free androgen index (FAI), and the testosterone/estradiol ratio (T/E_2_) [11]. The findings indicated that initially WC increases were associated with a rise in testosterone levels among children and adolescents, which subsequently declined. In male children, an increase in WC was positively correlated with estradiol levels and associated with a decrease in SHBG levels. These results underscore the significance of WC as a crucial biomarker in assessing hormone levels in children and adolescents.

Although there is an extensive research on obesity, studies exploring the relationship between WC and male adult hormone levels are limited, with most research confined to small, nonrepresentative samples [12, 13]. Using data from the National Health and Nutrition Examination Survey (NHANES), we investigated the associations between obesity (measured by WC), sex hormones (testosterone and estradiol), and sex hormone-binding globulin (SHBG). Considering the significant health risks associated with obesity and the dysregulation of steroid hormones, understanding the link between WC and hormone imbalance is crucial for developing effective public health policies.

## 2. Materials and Methods

### 2.1. Study Design and Participants

**Figure 1:**
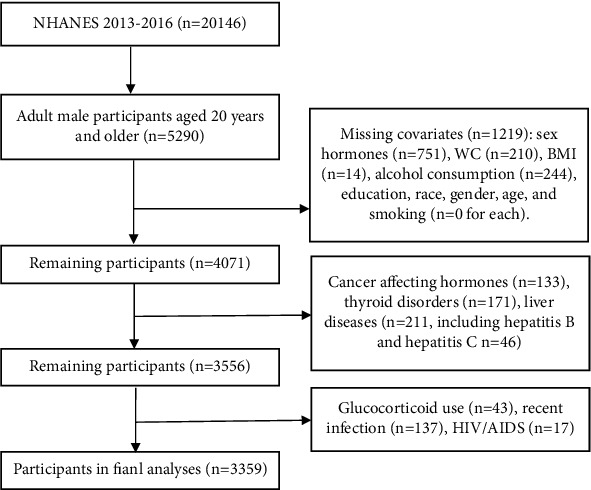
Flowchart for selecting the study population. NHANES: the National Health and Nutrition Examination Survey.

The National Health and Nutrition Examination Survey (NHANES) is a series of cross-sectional studies conducted in the United States aimed at evaluating the health and nutritional status of both adults and children. Unlike other studies, NHANES utilizes both physical examinations and interviews to gather comprehensive data. The National Center for Health Statistics Ethics Review Board reviewed and approved each study component, while each participant gave their written informed consent [11]. For our study, we included 3,359 adult men over the age of 20 years with data on WC, serum total testosterone (TT), estradiol (E_2_), and sex hormone-binding globulin (SHBG) from the 2013–2016 NHANES. Our selection process is shown in Figure 1.

### 2.2. Measurements of Sex Hormone Indicators

The sex hormone indicators utilized in this study included total testosterone (TT), estradiol (E_2_), sex hormone-binding globulin (SHBG), testosterone/estradiol ratio (T/E_2_), and free androgen index (FAI). Before the analysis at the National Center for Environmental Health, serum samples were prepared and stored in vials at −20°C. The concentrations of SHBG were measured using immuno-antibodies and chemiluminescence assays with a photomultiplier tube. The concentrations of TT and E_2_ were measured using isotope dilution high-performance liquid chromatography-tandem mass spectrometry (ID-LC-MS/MS). To indirectly estimate the levels of circulating free androgens and aromatase activity, we standardized the units of total testosterone and estradiol to nanomoles per liter (nmol/L), ensuring accuracy in our calculations. We used the following conversion formulas: total testosterone: 1 ng/dL = 0.0347 nmol/L; estradiol: 1 pg/mL = 0.003671 nmol/L. We then calculated the free androgen index (FAI) and the testosterone/estradiol ratio (T/E_2_): FAI = [(Total Testosterone (nmol/L) × 100)/Sex Hormone Binding Globulin (SHBG) (nmol/L)]; T/E_2_ = [Total Testosterone (nmol/L)/Estradiol (nmol/L)].

### 2.3. Measurements of Waist Circumference and BMI

Trained personnel followed standardized procedures for measuring the weight, height, and WC of all participants aged 20 years or older. During the measurements, participants were asked to wear only underclothing and an examination gown, and they were weighed in mobile examination facilities. To measure WC, a horizontal line was drawn over the uppermost external edge of the right iliac bone. These measurements were taken to guarantee an accurate assessment of the participants' physical characteristics.

### 2.4. Covariates

The selection of covariates in this study was informed by previous research on sex hormones [11, 13–15], which included age (continuous), race (Mexican American, other Hispanic, non-Hispanic White, non-Hispanic Black, non-Hispanic Asian, or other race), education level (less than 9th grade, 9−11th grade, high school graduate/GED or equivalent, some college or AA degree, or college graduate or above), poverty income ratio (PIR, continuous), body mass index (BMI, continuous), diabetes (categorical), session of blood sample collection (morning, afternoon, or evening), cotinine (continuous), alcohol intake (1–5 drinks/month, 5–10 drinks/month, 10+drinks/month, or nondrinker), smoking status (never smoker, former smoker, or current smoker), and physical activity (categorical).

Serum cotinine is quantified using an isotope-dilution high-performance liquid chromatography/atmospheric pressure chemical ionization tandem mass spectrometry (ID HPLC-APCI MS/MS). The *m*/*z* 80 product ion from the *m*/*z* 177 quasimolecular ion is specifically targeted for cotinine quantification. To identify participants with diabetes, the following criteria were applied: (a) hemoglobin A1C concentration ≥6.5% or a fasting plasma glucose level ≥126 mg/dL; (b) affirmative answers to any of the questions: “Take diabetic pills to lower blood sugar?” “Doctor told you have diabetes?” or “Taking insulin now? [16].” Participants who currently smoke or have smoked more than 100 cigarettes in their lifetime are identified as current or former smokers, respectively [17].

### 2.5. Statistical Analyses

We used sampling weights to adjust for selection probabilities, oversampling, nonresponse, and demographic discrepancies between the sample and the entire US population. Due to the right-skewed distribution of sex hormone indicators, we applied a log_2_ transformation to SHBG, while testosterone, estradiol, FAI, and T/E_2_ underwent square root transformation.

The Spearman correlation coefficient was employed to examine the correlation between WC and sex steroid hormones. The weighted univariate linear regression was employed to ascertain the correlations between WC and sex steroid hormones. We used weighted multivariable linear regression models to examine the relationship between WC and sex hormones, including adjustments for various confounding factors such as age, race, education level, poverty income ratio, diabetes, session of blood sample collection, cotinine, alcohol intake, smoking status, and physical activity. The Spearman correlation coefficient between WC and BMI is 0.91, suggesting a high degree of collinearity. Consequently, we omitted the BMI as a confounder to avoid the impact of multicollinearity on the multiple linear regression model. In this study, we adopted the generalized additive model (GAM) for detecting the nonlinear association. If the relationship between WC and sex hormones proved nonlinear, a two-piecewise linear regression model was applied to estimate the threshold effect. Subgroup analyses were performed using stratified linear regression models, and the modifications and interactions within subgroups were assessed through the likelihood ratio test. We analyzed the data using R software (version 4.2.2) and EmpowerStats (https://www.empowerstats.com). We considered a *P* value of <0.05 as statistically significant.

## 3. Results

### 3.1. Baseline Characteristics

The demographic and clinical characteristics of the study population are presented in Table 1. The study included 3,359 adult men from the United States, with an average age of 47.41 years (±17.29). The mean WC across the entire population was 100.76 cm (±15.71), showing a steady increase from Q1 to Q4. The BMI increased significantly across quartiles (*P* < 0.001), with the highest mean BMI observed in Q4. The prevalence of obesity in classes I, II, and III was notably higher in the WC third and fourth quartiles (Q3 and Q4). Total testosterone, SHBG, FAI, and the T/E_2_ ratio all showed marked decreases as WC increased (*P* < 0.001). Concurrently, total estradiol levels varied significantly across WC categories, with the highest levels observed in the group with the largest WC. Other demographic and lifestyle factors, including age, cotinine levels, poverty income ratio, racial distribution, educational attainment, diabetes prevalence, duration of physical activity, alcohol consumption, and smoking status, showed significant disparities across quartiles (*P* < 0.05). Detailed results of pairwise comparisons for variables with significant differences can be found in Supplementary Table 1.

### 3.2. Scatter Plots and Correlation Coefficients

Supplementary Figure 1 illustrates the correlations between WC and sex hormones. The Spearman correlation test revealed varying degrees of correlation between WC and sex hormones. Specifically, WC demonstrated a moderate negative correlation with testosterone (Spearman's rho = −0.434, *P* < 0.001) and the T/E_2_ ratio (Spearman's rho = −0.535, *P* < 0.001). A mild positive correlation was noted with estradiol (Spearman's rho = 0.108, *P* < 0.001). In addition, mild negative correlations were detected with SHBG (Spearman's rho = −0.189, *P* < 0.001) and FAI (Spearman's rho = −0.181, *P* < 0.001).

### 3.3. Weighted Univariate Analysis

This study investigated the impact of various factors on sex steroid hormone concentrations (Supplementary Table 2). Age showed a negative association with testosterone, FAI, and the T/E_2_ ratio, while it was positively associated with SHBG. Notably, both WC and BMI were found to have negative correlations with testosterone, SHBG, FAI, and the T/E_2_ ratio but a positive correlation with estradiol, particularly in individuals with larger WC and higher obesity classes. Cotinine levels demonstrated a positive correlation with both testosterone and SHBG, suggesting that higher nicotine exposure might be linked to increased levels of these hormones. The poverty income ratio was inversely correlated with both testosterone and the T/E_2_ ratio, while the diabetes status showed positive correlations with both estradiol and SHBG. Physical activity time, education level, alcohol intake, and smoking status exhibited variable associations with sex steroid hormones. These observations underscore the complexity of the relationships between lifestyle factors and hormonal profiles.

### 3.4. Association between Waist Circumference and Testosterone

**Figure 2:**
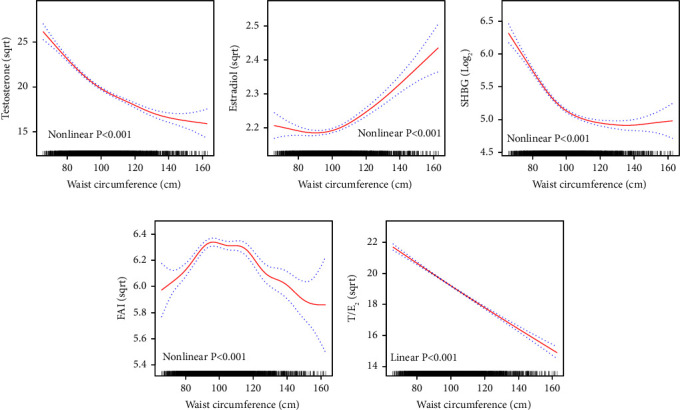
Curve fitting relationships between WC and sex steroid hormones. The solid and dashed lines in the graph represent the estimated values and corresponding 95% confidence intervals of transformed sex steroid hormones, respectively. WC: waist circumference; SHBG: sex hormone-binding globulin; T/E_2_: testosterone/estradiol; FAI: free androgen index. Curves adjusted for age, race, education, poverty income ratio, diabetes, sample collection session, cotinine, physical activity, alcohol, and smoking. Linear/nonlinear *P* values are indicated in the graph.

After controlling for potential confounding factors, the multivariate linear regression analysis confirmed an inverse relationship between WC and testosterone, consistently observed across all models (Table 2). The fully adjusted model demonstrated a significant negative trend with increasing quartiles of WC (*P* for trend <0.001). For every 1 cm increase in WC, the square root of testosterone is reduced by 0.117 units (*β* = −0.117, *P* < 0.001). The smoothing curve fitting graph (Figure 2) and threshold effect analysis (Supplementary Table 3) indicate a turning point at a WC of 97.6 cm. A steeper negative correlation occurs when the WC is less than or equal to this value (*β* = −0.190, *P* < 0.001). The effect of WC on testosterone levels varies significantly across different racial groups, BMI categories, education levels, and the diabetic population (Supplementary Table 4). Surprisingly, underweight individuals exhibited a positive association between WC and testosterone (*β* = 0.351, *P* = 0.016), while all other BMI groups demonstrated a negative association. Lifestyle factors, including physical activity, alcohol intake, and smoking status, did not significantly alter the relationship between WC and testosterone levels.

### 3.5. Association between Waist Circumference and Estradiol

In the fully adjusted multivariate linear regression model, a positive correlation was observed between WC and estradiol (Table 2, *β* = 0.002, *P*=0.002), especially in the highest quartile of WC (*β* = 0.077, *P*=0.014). The threshold effect analysis (Supplementary Table 3) identified an inflection point at 104.5 cm (Figure 2), after which the association became significantly positive (*β* = 0.004, *P* < 0.001). The results of the stratified analysis show that the association between WC and estradiol levels varies by race, BMI, educational attainment, timing of blood sample collection, and alcohol intake (Supplementary Table 4).

### 3.6. Association between Waist Circumference and SHBG

In the multivariate linear regression models, a consistent negative correlation between WC and SHBG levels was observed, intensifying across increasing quartiles (Table 2, *P* for trend <0.001). The smoothing curve fitting graph (Figure 2) and threshold effect analysis (Supplementary Table 3) identified a critical inflection point at 99.1 cm. Before reaching this inflection point, the log2 of SHBG decreases by 0.036 units for every 1 cm increase in WC (*β* = −0.036, *P* < 0.001). Beyond this point, the decrease in SHBG levels becomes significantly less steep (*β* = −0.004, *P* < 0.001). Furthermore, the stratified analysis in Supplementary Table 4 indicates that the relationship between WC and SHBG levels varies across different subgroups of US adult men.

### 3.7. Association between Waist Circumference and FAI

The fully adjusted multivariate linear regression model demonstrated no significant trend in FAI across increasing quartiles of WC (Table 2, *P* for trend = 0.213). The analysis using a smoothing curve-fitting graph (Figure 2) and threshold effect analysis (Supplementary Table 3) identified an inflection point at 98.6 cm. Below or at this point, a positive association (*β* = 0.013, *P* < 0.001) was observed, while beyond this point, a negative association (*β* = −0.009, *P* < 0.001) was evident. Subgroup analyses in Supplementary Table 4 indicate that the patterns of association between WC and FAI varied across different population groups.

### 3.8. Association between Waist Circumference and T/E_2_

The fully adjusted model demonstrated a significant negative trend in the T/E_2_ ratio with increasing WC (Table 2, *β* = −0.074, *P* < 0.001). The threshold effect model, however, did not provide a significantly better fit than the linear model (*P*=0.069), indicating no significant shift in the relationship between WC and the T/E_2_ ratio around 129.6 cm (Figure 2 and Supplementary Table 3). In addition, subgroup analyses in adult men revealed varied patterns in the association between WC and the T/E_2_ ratio, significantly influenced by factors such as race, session of blood sample collection, and smoking status (Supplementary Table 4).

## 4. Discussion

Obesity represents a growing global public health concern [1]. Traditionally, the body mass index (BMI) has served to assess obesity; however, it fails to account for the distribution of body fat and is affected by changes in muscle mass, making it an inadequate tool alone for evaluating obesity-related health risks [18]. Generally, individuals over 60 years or those who are chronically physically inactive might experience a decrease in skeletal muscle mass while maintaining or even reducing their BMI, whereas visceral adipose tissue accumulates, resulting in an increased WC [3, 19]. Research indicates that WC is on the rise among both children and adults, outpacing BMI trends [18]. Recent consensus identifies WC as a more precise indicator of visceral fat and a critical measure for assessing obesity-related health risks [3]. Therefore, it is crucial for health professionals to routinely incorporate WC measurements in clinical practice, considering it a necessary “vital sign” [3].

Our study observed a gradual decrease in serum testosterone levels as WC increased, consistent with prior cross-sectional studies [12, 13]. Kelly et al. conducted a review which showed that obesity is negatively associated with total testosterone levels across all age groups [20]. Adipocyte aromatase, found in abdominal adiposity, converts testosterone to estradiol, which serves as a negative feedback mechanism on the hypothalamic-pituitary (HP) axis, inhibiting gonadotropin-releasing hormone (GnRH) and reducing gonadal testosterone release. This interdependent relationship between low serum testosterone levels and obesity forms the classic hypogonadism-obesity cycle [20]. It is crucial to note that declining serum testosterone levels are not necessarily an inevitable consequence of aging. Instead, these decreases are often associated with opioid usage, chronic diseases linked to obesity, and modifiable health-related behaviors [19]. Obesity has also been causally linked to depression, whereby some subtypes of depression potentially decrease testosterone levels [21, 22]. Patients with obesity may be taking medications such as opioids and, to a lesser degree, statins, which lower serum testosterone concentrations [19]. According to Lopez et al., among obese men, long-term adherence to the prudent dietary pattern may lead to higher testosterone levels by lowering the BMI [23]. Regular aerobic exercise, particularly at higher intensities, is associated with modest reductions in WC and visceral adipose tissue and may provide more significant health benefits compared to moderate-intensity exercise [2]. Therefore, by adopting reasonable dietary control and engaging in necessary exercise, adult men can reduce their WC, decrease their body weight, and increase their serum testosterone concentrations. Such strategies have significant implications for the prevention and treatment of obesity-related hypogonadism in men.

In our study, we observed an intriguing correlation: among underweight individuals, WC positively correlates with testosterone levels (*β* = 0.351, *P*=0.016), although this correlation does not extend to weight groups of adult males. This phenomenon may be linked to increased levels of leptin and decreased lipolysis in subcutaneous fat. Leptin, a hormone secreted by fat cells, not only regulates body weight and food intake but also stimulates gonadotropin-releasing hormone (GnRH) neurons in the hypothalamus, leading to the release of luteinizing hormone and subsequent testosterone secretion by the testes [20]. We hypothesize that in underweight individuals, an increase in WC might be accompanied by an increase in fat cell numbers, thus elevating leptin levels and enhancing testosterone production [24]. In addition, testosterone can reduce lipolysis in subcutaneous fat by downregulating hormone-sensitive lipase and *β*2-adrenergic receptor expression [25]. Consequently, despite overall low body weight, underweight individuals may exhibit increased fat accumulation in specific areas, such as the abdomen, leading to a relative increase in WC. Future studies should further explore the interaction between WC and testosterone and their underlying mechanisms.

Our study identified a positive correlation between WC and estradiol levels, particularly evident when WC exceeds 104.5 cm. Similar observations have been made in male children [11] and adults [13]. In obese men, elevated estradiol levels occur due to increased aromatase activity, which converts more testosterone to estrogen. This increase in estrogen triggers a negative feedback mechanism in the hypothalamus, inhibiting GnRH release, and subsequently reducing LH and FSH levels, further disrupting hormone balance [6, 20]. To improve male reproductive health, addressing obesity is crucial. Key strategies include enhancing nutrition, increasing exercise, considering micronutrient and phototherapy supplements, and using medical interventions such as aromatase inhibitors and selective estrogen receptor modulators to balance testosterone and estrogen levels [26].

Our research confirmed a significant negative correlation between WC and SHBG levels in American adult males, mirroring findings across various age groups, including children and adolescents [11]. This trend highlights the broader epidemiological implications at various stages of male development. Meta-analyses indicate that bariatric surgery significantly increases total testosterone (TT) and SHBG levels, while decreasing estradiol levels in males, showcasing considerable hormonal shifts postoperation [27]. Furthermore, reports indicate that decreases in the subcutaneous adipose tissue (SAT) and visceral adipose tissue (VAT) are partly related to elevated TT in males and higher SHBG levels in both sexes [28]. In addition, AbbasiHormozi et al.'s study, which involved various groups, including controls (*n* = 40), obese (*n* = 40), lean-diabetic (*n* = 35), and obese-diabetic individuals (*n* = 35), found significantly lower levels of TT and SHBG in obese and diabetic men compared to controls [29]. These findings underscore the need for further research into the complex interactions between obesity and hormonal changes, particularly with a focus on the implications for male fertility and metabolic health.

The free androgen index (FAI), also known as the free testosterone index, is a method for estimating free testosterone levels in serum. It calculates the ratio by using total testosterone and SHBG (sex hormone-binding globulin) concentrations [30]. The simplicity of this method has led to its widespread adoption in clinical biochemistry laboratories. Despite its prevalent use, the accuracy of FAI in reflecting the true androgen status in males remains a topic of ongoing debate [31]. Studies have highlighted that FAI might not reliably estimate calculated free testosterone (CFT), particularly in males with low SHBG levels, where it tends to overestimate CFT. This overestimation may lead to misconceptions when evaluating male free testosterone levels [32]. However, the FAI continues to be extensively utilized not only in research [33] but also in the clinical investigation of hyperandrogenemia in females [34]. Overall, while the FAI remains a popular tool in hormonal studies, its effectiveness and reliability in various clinical contexts continue to be scrutinized, necessitating careful interpretation of results when used.

Our study demonstrated a significant negative correlation between the T/E_2_ ratio and WC, with the ratio decreasing as the WC increases (*β* = −0.074, *P* < 0.001). This finding aligns with the previously mentioned hypogonadism-obesity cycle. Specifically, adipocyte aromatase, prevalent in abdominal adiposity, converts testosterone into estradiol, thereby reducing the T/E_2_ ratio. Notably, this ratio is considered a more valuable and reliable indicator for assessing male infertility than the serum levels of either hormone alone [35]. In support of these conclusions, Maghsoumi-Norouzabad et al. conducted a cross-sectional study with 119 adult males, which revealed that the mean T/E_2_ ratio was significantly lower in obese infertile males than in those who are overweight and of normal weight, and also lower in males with a WC greater than 102 cm compared to those with a WC less than 102 cm [36]. Future studies should explore the applicability of the T/E_2_ ratio across different populations and investigate potential therapeutic interventions.

Our study possesses several strengths. Firstly, we obtained our data from the NHANES database, which ensured standardized measurements of WC and accurate serum hormone levels. We also weighted our data to better reflect the health status of adult men across the United States. Our comprehensive analysis incorporated a stratified approach, identifying specific demographics such as the underweight population, thus enhancing the utility of our findings for clinical references. However, our study has limitations. Despite adjusting for numerous potential confounders, unaccounted factors may still influence our results. In addition, our dataset lacks information on gonadotropin-releasing hormones, gonadotropins, and key enzymes, which limits our ability to delve into the biological mechanisms at play. Importantly, the cross-sectional nature of our study constrains us from establishing causality between obesity and sex steroid hormone levels. Another significant concern is the nonnormal distribution of testosterone, estradiol, and the FAI, despite our efforts to normalize the data. This issue might introduce bias into our interpretations and should be considered critically. Future research should consider employing alternative data transformation methods or nonparametric statistical techniques to overcome these limitations. Overall, this study provides insightful observations into the complex interplay between abdominal obesity and sex steroid hormones in adult men. To enhance the robustness of our conclusions, further prospective studies are warranted.

## 5. Conclusions

In conclusion, waist circumference negatively correlates with testosterone, SHBG, and T/E_2_ but positively correlates with estradiol while showing a positive correlation with testosterone in underweight individuals. Our study suggests that waist circumference should be a crucial anthropometric predictor of sex steroid hormone levels in adult men.

## Figures and Tables

**Table 1:** Characteristics of the population.

Characteristics	Total population	Total population^†^	WC Q1 (65.80 cm–89.70 cm)	WC Q2 (89.80 cm–99.40 cm)	WC Q3 (99.50 cm −109.30 cm)	WC Q4 (109.40 cm −162.70 cm)	*P* value
Number	3359	3359	836	843	840	840	
Age (year, mean ± SD)	47.41 (17.29)	45.34 (0.43)	38.74 (16.35)	48.40 (16.37)	51.67 (16.56)	50.79 (16.79)	<0.001
Early adulthood^‡^ (*N*, %)	1573 (46.83%)	50.32%	566 (67.70%)	367 (43.53%)	318 (37.86%)	322 (38.33%)	
Middle age (*N*, %)	1132 (33.70%)	34.60%	193 (23.09%)	319 (37.84%)	303 (36.07%)	317 (37.74%)	
Late life (*N*, %)	654 (19.47%)	15.07%	77 (9.21%)	157 (18.62%)	219 (26.07%)	201 (23.93%)	
BMI (kg/m^2^, mean ± SD)	28.66 (5.97)	28.84 (0.17)	22.72 (2.40)	26.42 (2.14)	29.38 (2.37)	36.12 (5.50)	<0.001
Underweight^§^ (*N*, %)	38 (1.13%)	1.17%	38 (4.55%)	0 (0.00%)	0 (0.00%)	0 (0.00%)	
Normal weight (*N*, %)	885 (26.35%)	24.45%	647 (77.39%)	223 (26.45%)	14 (1.67%)	1 (0.12%)	
Overweight (*N*, %)	1282 (38.17%)	38.62%	150 (17.94%)	571 (67.73%)	499 (59.40%)	62 (7.38%)	
Obesity class I (*N*, %)	717 (21.35%)	22.40%	1 (0.12%)	49 (5.81%)	312 (37.14%)	355 (42.26%)	
Obesity class II (*N*, %)	269 (8.01%)	8.51%	0 (0.00%)	0 (0.00%)	15 (1.79%)	254 (30.24%)	
Obesity class III (*N*, %)	168 (5.00%)	4.84%	0 (0.00%)	0 (0.00%)	0 (0.00%)	168 (20.00%)	
WC (cm, mean ± SD)	100.76 (15.71)	101.68 (0.49)	82.32 (5.47)	94.74 (2.75)	104.33 (2.82)	121.60 (10.93)	<0.001
Total testosterone (ng/dl, mean ± SD)	422.94 (181.68)	425.99 (4.08)	535.34 (208.96)	436.92 (165.53)	388.14 (137.76)	331.86 (140.98)	<0.001
Total estradiol (pg/ml, mean ± SD)	25.03 (9.77)	24.97 (0.29)	24.95 (10.63)	23.96 (9.28)	24.10 (7.93)	27.10 (10.68)	<0.001
SHBG (nmol/l, median, Q1-Q3)	37.53 (26.82–53.29)	36.81 (26.53–51.85)	43.16 (31.38–61.46)	37.37 (26.52–54.58)	37.09 (26.66–51.99)	33.09 (24.25–46.67)	<0.001
FAI (median, Q1-Q3)	35.92 (26.34–48.82)	40.93 (39.90, 41.96)	40.32 (29.94–53.66)	37.98 (27.44–51.09)	34.40 (25.35–46.58)	31.53 (24.07–43.13)	<0.001
T/E_2_ (median, Q1-Q3)	158.73 (119.90–208.64)	173.29 (167.76, 178.82)	204.31 (162.86–259.41)	170.90 (137.30–222.13)	151.85 (120.98–189.63)	114.03 (86.93–150.45)	<0.001
Cotinine (median, Q1-Q3)	0.05 (0.01–56.80)	0.04 (0.01–39.90)	0.11 (0.02–155.00)	0.05 (0.01–31.85)	0.03 (0.01–23.15)	0.04 (0.01–19.25)	<0.001
Poverty income ratio (median, Q1-Q3)	2.02 (0.95–3.97)	2.89 (1.28–5.00)	1.82 (0.86–3.57)	2.02 (0.98–4.25)	2.01 (0.98–4.04)	2.12 (1.07–3.90)	0.017
Race							<0.001
Mexican American (*N*, %)	543 (16.17%)	9.81%	88 (10.53%)	146 (17.32%)	167 (19.88%)	142 (16.90%)	
Other Hispanic (*N*, %)	345 (10.27%)	6.00%	66 (7.89%)	103 (12.22%)	103 (12.26%)	73 (8.69%)	
Non-Hispanic white (*N*, %)	1320 (39.30%)	66.27%	265 (31.70%)	295 (34.99%)	349 (41.55%)	411 (48.93%)	
Non-Hispanic black (*N*, %)	653 (19.44%)	9.80%	204 (24.40%)	140 (16.61%)	141 (16.79%)	168 (20.00%)	
Non-Hispanic Asian (*N*, %)	381 (11.34%)	5.01%	180 (21.53%)	131 (15.54%)	59 (7.02%)	11 (1.31%)	
Other race (*N*, %)	117 (3.48%)	3.10%	33 (3.95%)	28 (3.32%)	21 (2.50%)	35 (4.17%)	
Education level							<0.001
Less than 9th grade (*N*, %)	306 (9.11%)	4.75%	54 (6.46%)	95 (11.27%)	93 (11.07%)	64 (7.62%)	
9−11th grade (*N*, %)	444 (13.22%)	9.78%	115 (13.76%)	124 (14.71%)	105 (12.50%)	100 (11.90%)	
High school graduate/GED or equivalent (*N*, %)	796 (23.70%)	22.85%	187 (22.37%)	196 (23.25%)	195 (23.21%)	218 (25.95%)	
Some college or AA degree (*N*, %)	937 (27.90%)	29.63%	241 (28.83%)	182 (21.59%)	226 (26.90%)	288 (34.29%)	
College graduate or above (*N*, %)	876 (26.08%)	32.98%	239 (28.59%)	246 (29.18%)	221 (26.31%)	170 (20.24%)	
Diabetes							<0.001
Yes (*N*, %)	579 (17.24%)	13.49%	47 (5.62%)	124 (14.71%)	152 (18.10%)	256 (30.48%)	
No (*N*, %)	2780 (82.76%)	86.51%	789 (94.38%)	719 (85.29%)	688 (81.90%)	584 (69.52%)	
Session of blood sample collection							0.258
Morning (*N*, %)	1601 (47.66%)	48.38%	391 (46.77%)	417 (49.47%)	417 (49.64%)	376 (44.76%)	
Afternoon (*N*, %)	1239 (36.89%)	35.31%	305 (36.48%)	301 (35.71%)	309 (36.79%)	324 (38.57%)	
Evening (*N*, %)	519 (15.45%)	16.32%	140 (16.75%)	125 (14.83%)	114 (13.57%)	140 (16.67%)	
Physical activity time (hour/week)							0.013
Nonactivity	1907 (56.77%)	52.06%	491 (58.73%)	496 (58.84%)	481 (57.26%)	439 (52.26%)	
0.1–0.9 (*N*, %)	102 (3.04%)	3.16%	24 (2.87%)	22 (2.61%)	25 (2.98%)	31 (3.69%)	
1.0–3.4 (*N*, %)	297 (8.84%)	10.46%	55 (6.58%)	80 (9.49%)	75 (8.93%)	87 (10.36%)	
3.5–5.9 (*N*, %)	125 (3.72%)	4.28%	20 (2.39%)	38 (4.51%)	36 (4.29%)	31 (3.69%)	
≥6 (*N*, %)	928 (27.63%)	30.04%	246 (29.43%)	207 (24.56%)	223 (26.55%)	252 (30.00%)	
Alcohol intake^¶^							0.039
Nondrinker (*N*, %)	592 (17.62%)	14.38%	175 (20.93%)	148 (17.56%)	130 (15.48%)	139 (16.55%)	
1–5 drinks/month (*N*, %)	1816 (54.06%)	51.36%	421 (50.36%)	453 (53.74%)	454 (54.05%)	488 (58.10%)	
5–10 drinks/month (*N*, %)	331 (9.85%)	11.25%	85 (10.17%)	83 (9.85%)	84 (10.00%)	79 (9.40%)	
10+ drinks/month (*N*, %)	620 (18.46%)	23.01%	155 (18.54%)	159 (18.86%)	172 (20.48%)	134 (15.95%)	
Smoking status							<0.001
Never smoker (*N*, %)	1643 (48.91%)	51.81%	452 (54.07%)	416 (49.35%)	382 (45.48%)	393 (46.79%)	
Former smoker (*N*, %)	951 (28.31%)	28.21%	132 (15.79%)	246 (29.18%)	284 (33.81%)	289 (34.40%)	
Current smoker (*N*, %)	765 (22.77%)	19.98%	252 (30.14%)	181 (21.47%)	174 (20.71%)	158 (18.81%)	

^†^Applied sampling weights. In this column, values following the mean denote standard error (SE) and not standard deviation (SD); ^‡^early adulthood: 20–44 years, middle age: 45–64 years, and late life: ≥65 years. ^§^Underweight (BMI values <18.5); normal weight (BMI values 18.5–24.9); overweight (BMI values 25.0–29.9); obese class I (BMI values 30.0–34.9); obese class II (BMI values 35.0–39.9); obese class III (BMI values ≥ 40.0). ^¶^In this context, a “drink” refers to any of the following: a 12 oz serving of beer, a 5 oz glass of wine, or 1.5 oz of liquor. WC: waist circumference; BMI: body mass index; SHBG: sex hormone-binding globulin; T/E_2_: testosterone/estradiol; FAI: free androgen index.

**Table 2:** Association between sex hormones and waist circumference in adult males.

Waist circumference	Nonadjusted model *β*, 95% CI, *P*	Minimally adjusted model *β*, 95% CI, *P*	Fully adjusted model *β*, 95% CI, *P*
Exposure to testosterone	−0.117 (−0.127, −0.107) <0.001	−0.120 (−0.131, −0.108) <0.001	−0.117 (−0.136, −0.098) <0.001
Q1 (65.80 cm–89.70 cm)	Reference	Reference	Reference
Q2 (89.80 cm–99.40 cm)	−1.899 (−2.398, −1.401) <0.001	−1.956 (−2.464, −1.449) <0.001	−1.884 (−3.006, −0.761) 0.005
Q3 (99.50 cm −109.30 cm)	−3.152 (−3.593, −2.710) <0.001	−3.269 (−3.747, −2.791) <0.001	−3.215 (−4.339, −2.090) 0.001
Q4 (109.40 cm −162.70 cm)	−4.680 (−5.056, −4.304) <0.001	−4.849 (−5.229, −4.468) <0.001	−4.651 (−5.695, −3.606) <0.001
*P* for trend	<0.001	<0.001	<0.001
Exposure to estradiol	0.002 (0.001, 0.003) <0.001	0.002 (0.002, 0.003) <0.001	0.002 (0.001, 0.003) 0.002
Q1 (65.80 cm–89.70 cm)	Reference	Reference	Reference
Q2 (89.80 cm–99.40 cm)	0.000 (−0.027, 0.027) 0.992	0.006 (−0.020, 0.033) 0.650	0.007 (−0.052, 0.065) 0.647
Q3 (99.50 cm–109.30 cm)	0.009 (−0.011, 0.030) 0.378	0.017 (−0.006, 0.039) 0.168	0.016 (−0.035, 0.067) 0.277
Q4 (109.40 cm–162.70 cm)	0.071 (0.045, 0.097) <0.001	0.077 (0.050, 0.103) <0.001	0.077 (0.013, 0.140) 0.014
*P* for trend	<0.001	<0.001	0.002
Exposure to SHBG	−0.009 (−0.012, −0.007) <0.001	−0.016 (−0.018, −0.014) <0.001	−0.016 (−0.019, −0.012) <0.001
Q1 (65.80 cm–89.70 cm)	Reference	Reference	Reference
Q2 (89.80 cm–99.40 cm)	−0.160 (−0.250, −0.069) 0.002	−0.402 (−0.488, −0.316) <0.001	−0.394 (−0.597, −0.192) 0.004
Q3 (99.50 cm–109.30 cm)	−0.225 (−0.316, −0.135) <0.001	−0.568 (−0.658, −0.479) <0.001	−0.552 (−0.764, −0.341) 0.002
Q4 (109.40 cm–162.70 cm)	−0.392 (−0.483, −0.301) <0.001	−0.739 (−0.830, −0.649) <0.001	−0.715 (−0.939, −0.491) 0.001
*P* for trend	<0.001	<0.001	<0.001
Exposure to FAI	−0.008 (−0.010, −0.006) <0.001	−0.0014(-0.0033, 0.0005) 0.171	−0.0014 (−0.0041, 0.0013) 0.213
Q1 (65.80 cm–89.70 cm)	Reference	Reference	Reference
Q2 (89.80 cm–99.40 cm)	−0.097 (−0.186, −0.008) 0.043	0.157 (0.067, 0.247) 0.003	0.159 (−0.036, 0.353) 0.069
Q3 (99.50 cm–109.30 cm)	−0.215(-0.289, −0.142) <0.001	0.138 (0.068, 0.207) <0.001	0.129 (−0.029, 0.287) 0.059
Q4(109.40 cm–162.70 cm)	−0.299 (−0.383, −0.215) <0.001	0.052 (−0.031, 0.136) 0.230	0.059 (−0.124, 0.242) 0.262
*P* for trend	<0.001	0.171	0.213
Exposure to T/E_2_	−0.073 (−0.078, −0.069) <0.001	−0.076(-0.081, −0.071) <0.001	−0.074 (−0.082, −0.069) <0.001
Q1 (65.80 cm–89.70 cm)	Reference	Reference	Reference
Q2 (89.80 cm–99.40 cm)	−0.887 (−1.126, −0.648) <0.001	−0.959 (−1.215, −0.703) <0.001	−0.932 (−0.150, −0.354) 0.006
Q3 (99.50 cm–109.30 cm)	−1.574 (−1.782, −1.366) <0.001	−1.683 (−1.940, −1.426) <0.001	−1.650 (−2.192, −1.108) <0.001
Q4 (109.40 cm −162.70 cm)	−2.812 (−3.017, −2.607) <0.001	−2.933 (−3.147, −2.718) <0.001	−2.833 (−3.333, −2.332) <0.001
*P* for trend	<0.001	<0.001	<0.001

SHBG: sex hormone-binding globulin; T/E_2_: testosterone/estradiol; FAI: free androgen index. Nonadjusted model: no covariates were adjusted; minimally adjusted model: adjusted for age and race; fully adjusted model: adjusted for age, race, education level, poverty income ratio, diabetes, session of blood sample collection, cotinine, alcohol intake, smoking status, and physical activity. All models were weighted.

## Data Availability

All data generated or analyzed in this study were provided by the National Center for Health Statistics of the US Centers for Disease Control and Prevention (https://www.cdc.gov/nchs/nhanes/index.htm). For further data inquiries, please contact the corresponding author.

## Supplementary Materials

Supplementary Materials Supplementary Table 1: pairwise comparisons for significant differences among waist circumference groups. Supplementary Table 2: weighted univariate analysis for sex steroid hormones. Supplementary Table 3: threshold effect analysis of waist circumference on sex steroid hormones using piecewise linear regression models. Supplementary Table 4: stratified analysis of waist circumference and sex steroid hormones. Supplementary Figure 1: scatter plot of the relationship between WC and sex steroid hormones.
