# Supplementary figures and images for: Associations between Waist Circumference and Sex Steroid Hormones in US Adult Men: Cross-Sectional Findings from the NHANES 2013–2016
Source: Int J Endocrinol. 2024 Aug 26;2024:4306797. doi: 10.1155/2024/4306797 (PMC11368549; doi:10.1155/2024/4306797)

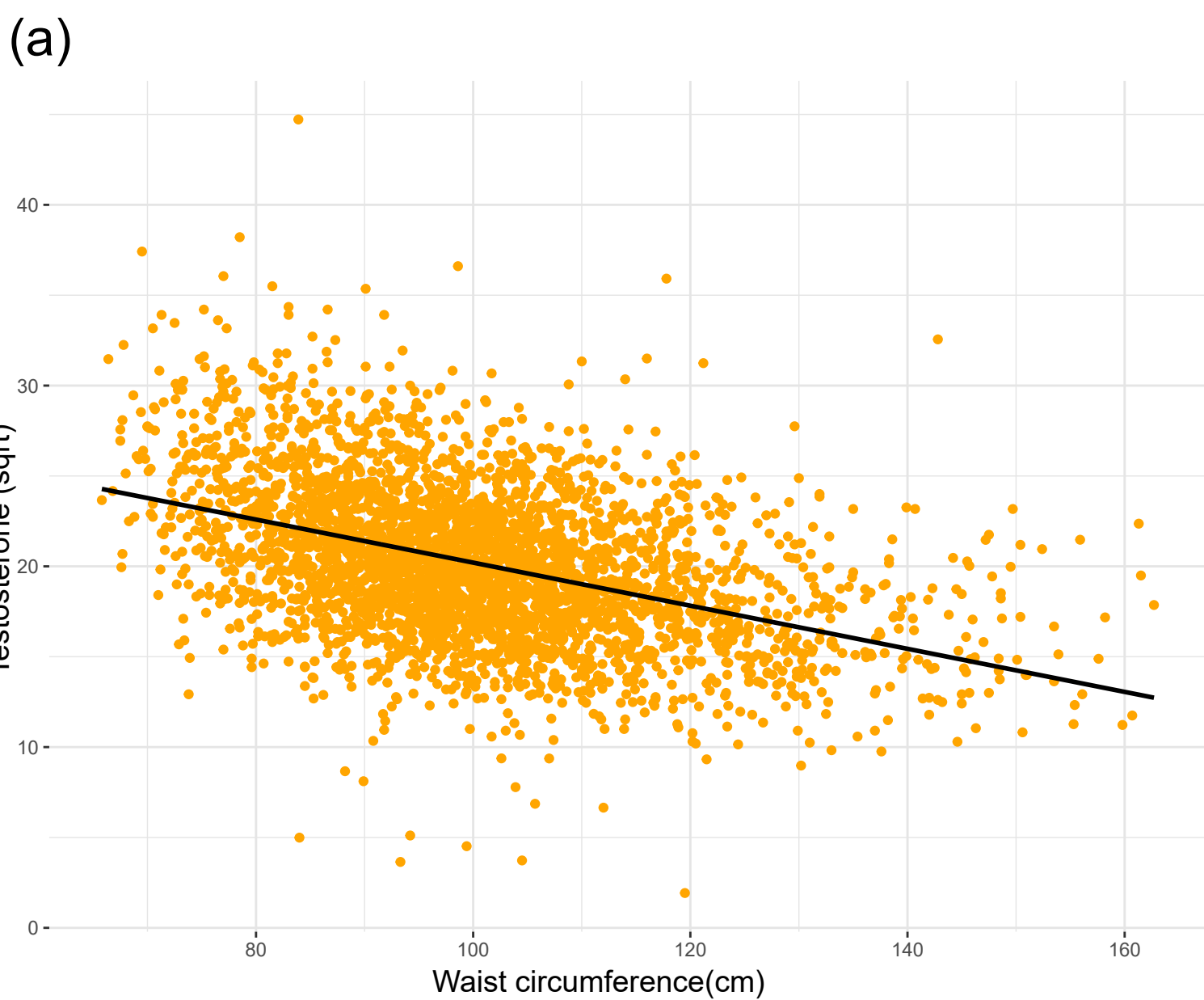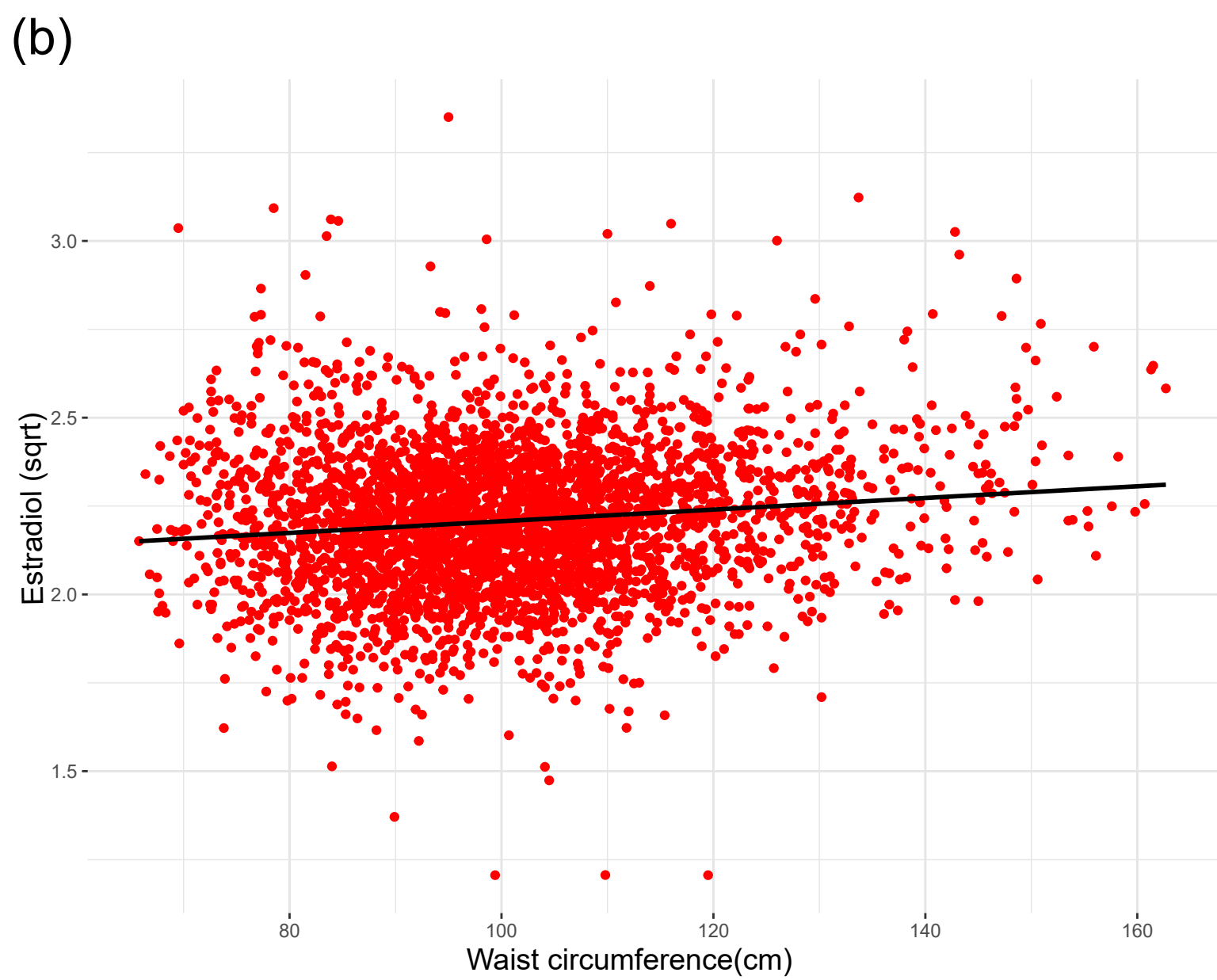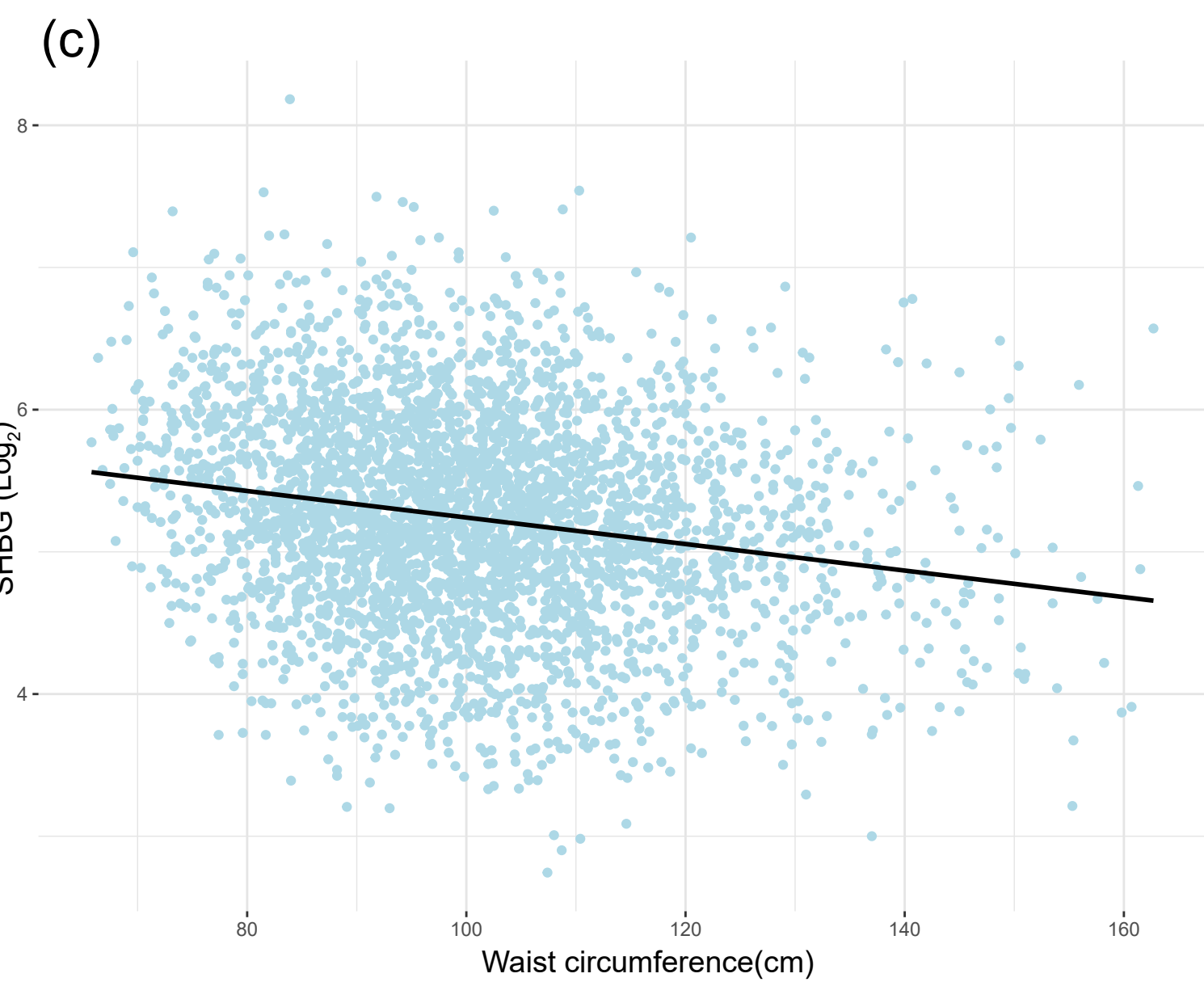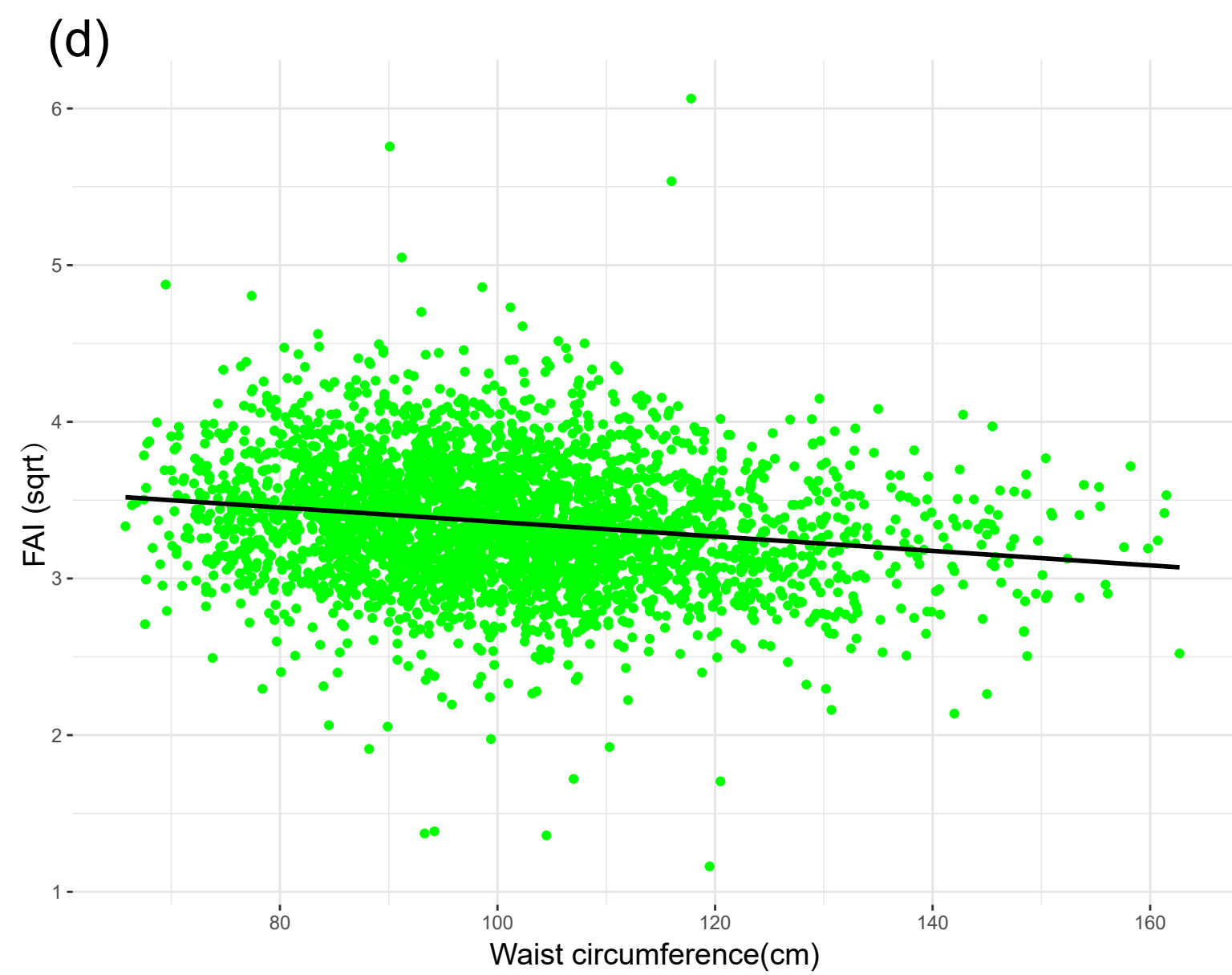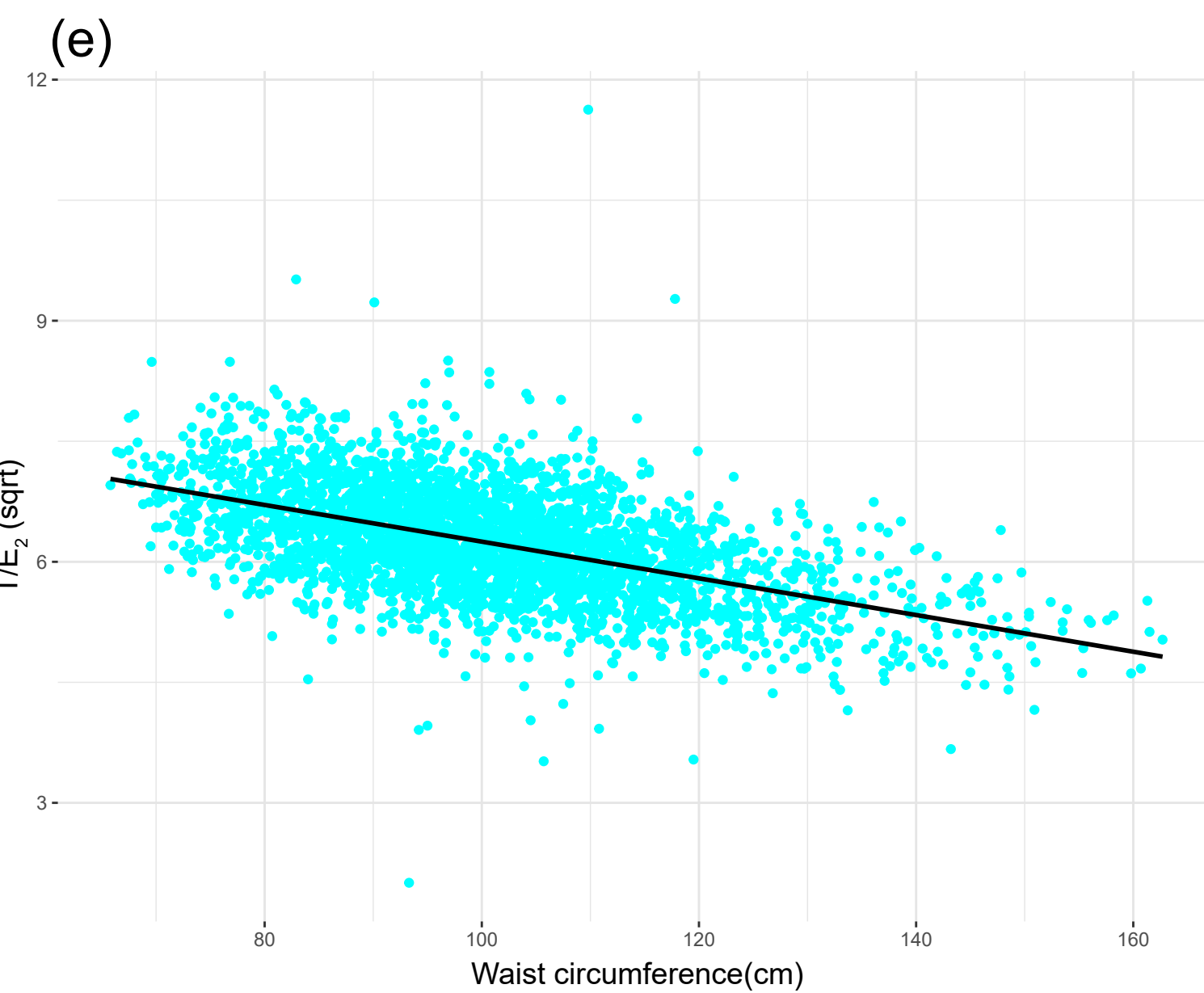

Supplement: Supplementary Materials — Supplementary Table 1: pairwise comparisons for significant differences among waist circumference groups. Supplementary Table 2: weighted univariate analysis for sex steroid hormones. Supplementary Table 3: threshold effect analysis of waist circumference on sex steroid hormones using piecewise linear regression models. Supplementary Table 4: stratified analysis of waist circumference and sex steroid hormones. Supplementary Figure 1: scatter plot of the relationship between WC and sex steroid hormones. [file 4306797.f1.zip › Supplementary Figure 1 (1).pdf]
