# Supplementary material for: Associations between Waist Circumference and Sex Steroid Hormones in US Adult Men: Cross-Sectional Findings from the NHANES 2013–2016
Source: Int J Endocrinol. 2024 Aug 26;2024:4306797. doi: 10.1155/2024/4306797 (PMC11368549; doi:10.1155/2024/4306797)
Supplement: Supplementary Materials — Supplementary Table 1: pairwise comparisons for significant differences among waist circumference groups. Supplementary Table 2: weighted univariate analysis for sex steroid hormones. Supplementary Table 3: threshold effect analysis of waist circumference on sex steroid hormones using piecewise linear regression models. Supplementary Table 4: stratified analysis of waist circumference and sex steroid hormones. Supplementary Figure 1: scatter plot of the relationship between WC and sex steroid hormones. [file 4306797.f1.zip › Supplementary Tables 1-4.doc]

Supplementary Tables 1-4

**Supplementary Table 1: Pairwise Comparisons for Significant Differences Among Waist Circumference Groups**

| Variable | Comparison | WC Q1 - WC Q2 | WC Q1 - WC Q3 | WC Q1 - WC Q4 | WC Q2 - WC Q3 | WC Q2 - WC Q4 | WC Q3 - WC Q4 |
| --- | --- | --- | --- | --- | --- | --- | --- |
| Age | Difference(year) | -9.66 | -12.94 | -12.05 | -3.28 | -2.39 | 0.89 |
|  | P-value | <0.001 | <0.001 | <0.001 | 0.0003 | 0.0159 | 0.6894 |
| WC | Difference(cm) | -12.42 | -22.01 | -39.28 | -9.59 | -26.86 | -17.27 |
|  | P-value | <0.001 | <0.001 | <0.001 | <0.001 | <0.001 | <0.001 |
| BMI | Difference(kg/m^2^) | -3.7 | -6.65 | -13.39 | -2.96 | -9.7 | -6.74 |
|  | P-value | <0.001 | <0.001 | <0.001 | <0.001 | <0.001 | <0.001 |
| PIR | Difference(median) | -0.2 | -0.17 | -0.24 | 0.04 | -0.03 | -0.07 |
|  | P-value | 0.0703 | 0.1878 | 0.0259 | 0.9723 | 0.9824 | 0.8509 |
| Cotinine | Difference(median) | 21.57 | 28.85 | 30.66 | 7.27 | 9.09 | 1.82 |
|  | P-value | 0.0116 | 0.0002 | 0.0001 | 0.7279 | 0.5659 | 0.9939 |
| Total Testosterone | Difference(ng/dl) | 98.42 | 147.2 | 203.48 | 48.78 | 105.06 | 56.28 |
|  | P-value | <0.001 | <0.001 | <0.001 | <0.001 | <0.001 | <0.001 |
| Total Estradiol | Difference(pg/ml) | 0.98 | 0.85 | -2.15 | -0.14 | -3.13 | -3 |
|  | P-value | 0.162 | 0.2803 | <0.001 | 0.9918 | <0.001 | <0.001 |
| SHBG | Difference(nmol/l) | 5.14 | 7.12 | 11.21 | 1.98 | 6.07 | 4.09 |
|  | P-value | <0.001 | <0.001 | <0.001 | 0.3017 | <0.001 | 0.0018 |
| FAI | Difference(median) | 0.75 | 1.61 | 2.4 | 0.86 | 1.64 | 0.78 |
|  | P-value | 0.0441 | <0.001 | <0.001 | 0.0144 | <0.001 | 0.0326 |
| T/E_2_ | Difference(median) | 3.51 | 5.92 | 9.56 | 2.42 | 6.05 | 3.63 |
|  | P-value | <0.001 | <0.001 | <0.001 | <0.001 | <0.001 | <0.001 |
| Race | P-value | <0.001 | <0.001 | <0.001 | <0.001 | 0.0002 | 0.9905 |
| Education level | P-value | 0.0342 | 0.0866 | 0.2252 | 0.9846 | 0.8535 | 0.9703 |
| Diabetes | P-value | <0.001 | <0.001 | <0.001 | 0.2321 | <0.001 | <0.001 |
| Physical activity | P-value | 0.8151 | 0.9994 | 0.3285 | 0.8716 | 0.0493 | 0.2674 |
| Alcohol intake | P-value | 0.8607 | 0.2087 | 0.9876 | 0.6498 | 0.6783 | 0.1029 |
| Smoking status | P-value | 0.7463 | 0.9966 | 0.732 | 0.8576 | 1 | 0.8461 |

WC:waist circumference; BMI: body mass index; PIR; Poverty income ratio; SHBG: sex hormone-binding globulin; T/E_2_: testosterone/estradiol; FAI: free androgen index.

**Supplementary Table 2: Weighted univariate analysis for sex steroid hormones**

| Covariate | Statistics | Testosterone β(95%CI) | Estradiol β(95%CI) | SHBG β(95%CI) | FAI β(95%CI) | T/E_2_ β(95%CI) |
| --- | --- | --- | --- | --- | --- | --- |
| Age (year, mean±SD) | 47.41 (17.29) | -0.028(-0.038, -0.017)** | 0.000(-0.000, 0.001) | 0.019(0.018, 0.020)** | -0.025(-0.027, -0.023)** | -0.017(-0.023, -0.011)** |
| Early Adulthood † (N,%) | 1573 (46.83%) | Reference | Reference | Reference | Reference | Reference |
| Middle Age (N,%) | 1132 (33.70%) | -0.842(-1.168, -0.515)** | 0.001(-0.011, 0.012) | 0.394 (0.349, 0.439)** | -0.570(-0.630, -0.511)** | -0.430(-0.599, -0.261)** |
| Late Life (N,%) | 654 (19.47%) | -0.767(-1.226, -0.308)* | 0.029(0.009, 0.049)* | 0.828 (0.745, 0.911)** | -1.002 (-1.081, -0.924)** | -0.643 (-0.882, -0.404)** |
| BMI(kg/m^2^, mean±SD) | 28.66 (5.97) | -0.294(-0.320, -0.268)** | 0.006(0.004, 0.008)** | -0.036(-0.041, -0.030)** | -0.008(-0.013, -0.003)* | -0.192 (-0.203, -0.180)** |
| Normal weight ‡ (N,%) | 885 (26.35%) | Reference | Reference | Reference | Reference | Reference |
| Underweight (N,%) | 38 (1.13%) | 2.525(0.845, 4.206)* | 0.035(-0.040, 0.111) | 0.336(0.146, 0.527)* | 0.013 (-0.218, 0.245) | -0.826 (-1.364, -0.288)* |
| Overweight (N,%) | 1282 (38.17%) | -2.287(-2.675, -1.898)** | -0.006(-0.026, 0.014) | -0.284(-0.362, -0.206)** | -0.011(-0.249, 0.227) | -1.825(-2.378, -1.272)** |
| Obesity Class I (N,%) | 717 (21.35%) | -3.230(-3.621, -2.838)** | 0.045(0.019, 0.071)* | -0.462(-0.577, -0.348)** | 0.046(-0.194, 0.286) | -2.725(-3.352, -2.097)** |
| Obesity Class II (N,%) | 269 (8.01%) | -4.893(-5.439, -4.348)** | 0.077(0.040, 0.115)** | -0.533(-0.660, -0.407)** | -0.184(-0.452, 0.085) | -3.839(-4.424, -3.254)** |
| Obesity Class III (N,%) | 168 (5.00%) | -6.117(-6.831, -5.404)** | 0.152(0.103, 0.201)** | -0.734(-0.862, -0.606)** | -0.186(-0.452, 0.081) | -4.980(-5.578, -4.381)** |
| WC (cm) | 100.76±15.71 | -0.117 (-0.127, -0.107)** | 0.002 (0.001, 0.003)** | -0.009 (-0.012, -0.007)** | -0.008 (-0.010, -0.006)** | -0.073(-0.078, -0.069)** |
| WC Q1 (cm) | 65.80 – 89.70 | Reference | Reference | Reference | Reference | Reference |
| WC Q2 (cm) | 89.80 – 99.40 | -1.890(-2.398, -1.401)** | 0.000(-0.027, 0.027) | -0.160(-0.250, -0.069)* | -0.097(-0.186, -0.008)* | -0.887 (-1.126, -0.648)** |
| WC Q3 (cm) | 99.50 -109.30 | -3.152(-3.593, -2.710)** | 0.009(-0.011, 0.030) | -0.225(-0.316, -0.135)** | -0.215(-0.289, -0.142)** | -1.575(-1.783, -1.366)** |
| WC Q4 (cm) | 109.40-162.70 | -4.680 (-5.056, -4.304)** | 0.071(0.045, 0.097)** | -0.392(-0.483, -0.301)** | -0.299(-0.383, -0.215)** | -2.812(-3.017, -2.607)** |
| Cotinine (median ,Q1-Q3) | 0.05 (0.01-56.80) | 0.002(0.001, 0.004)* | 0.000(-0.000, 0.000) | 0.000(0.000, 0.001)* | -0.000(-0.000, 0.000) | 0.001(0.000, 0.001)* |
| Poverty income ratio (median, Q1-Q3) | 2.02 (0.95-3.97) | -0.132(-0.248, -0.016)* | 0.000(-0.005, 0.005) | -0.004(-0.026, 0.018) | -0.014(-0.032, 0.003) | -0.068(-0.124, -0.012)* |
| Education level |  |  |  |  |  |  |
| Less than 9^th^ grade (N,%) | 306 (9.11%) | Reference | Reference | Reference | Reference | Reference |
| 9-11^th^ grade (N,%) | 444 (13.22%) | 0.171(-0.505, 0.848) | 0.061(0.015, 0.106)* | -0.098(-0.212, 0.016) | 0.141(0.021, 0.262)* | -0.439(-0.881, 0.002) |
| High school graduate/ GED or equivalent(N,%) | 796 (23.70%) | -0.098(-0.865, 0.668) | 0.057(0.023, 0.090)* | -0.086(-0.210, 0.038) | 0.082(-0.049, 0.213) | -0.529(-0.902, -0.157)* |
| Some college or AA degree (N,%) | 937 (27.90%) | -0.417(-1.129, 0.296) | 0.061(0.031, 0.091)** | -0.197(-0.329, -0.064)* | 0.157(0.024, 0.289)* | -0.727(-1.127, -0.327)** |
| College graduate or above (N,%) | 876 (26.08%) | -0.228(-0.995, 0.539) | 0.041(0.011, 0.071)* | -0.134(-0.268, 0.000)* | 0.126(-0.004, 0.256) | -0.448(-0.815, -0.082)* |
| Diabetes |  |  |  |  |  |  |
| No (N,%) | 2780 (82.76%) | Reference | Reference | Reference | Reference | Reference |
| Yes (N,%) | 579 (17.24%) | -1.630(-2.122, -1.139)** | 0.037(0.013, 0.061)* | 0.096(0.006, 0.187)* | -0.369(-0.485, -0.253)** | -1.118(-1.328, -0.906)** |
| Session of blood sample collection | |  |  |  |  |  |
| Morning (N,%) | 1601 (47.66%) | Reference | Reference | Reference | Reference | Reference |
| Afternoon (N,%) | 1239 (36.89%) | -1.692(-2.074, 1.310)** | -0.063(-0.081, -0.045)** | -0.063(-0.119, -0.006)* | -0.194(-0.267, -0.121)** | -0.246 (-0.410, -0.082)* |
| Evening (N,%) | 519 (15.45%) | -2.034(-2.578, -1.491)** | -0.074(-0.096, -0.053)** | -0.099(-0.169, -0.030)* | -0.227 (-0.297, -0.158)** | -0.321(-0.655, 0.014) |
| Physical activity time (hour / week) | |  |  |  |  |  |
| Non-activity | 1907 (56.77%) | Reference | Reference | Reference | Reference | Reference |
| 0.1-0.9 (N,%) | 102 (3.04%) | -0.876(-1.821, 0.069) | -0.041(-0.077, -0.004)* | 0.045(-0.153, 0.243) | -0.206(-0.432, 0.020) | -0.134(-0.645, 0.377) |
| 1.0-3.4 (N,%) | 297 (8.84%) | -0.362(-1.006, 0.283) | -0.016(-0.051, 0.020) | 0.031(-0.108, 0.170) | -0.081(-0.212, 0.050) | -0.035(-0.368, 0.298) |
| 3.5-5.9 (N,%) | 125 (3.72%) | -0.957(-1.669, -0.246)* | -0.036(-0.079, 0.007) | -0.059(-0.176, 0.058) | -0.077(-0.198, 0.044) | -0.117(-0.607, 0.372) |
| ≥6 (N,%) | 928 (27.63%) | 0.240(-0.204, 0.684) | -0.017(-0.037, 0.003) | -0.024(-0.113, 0.065) | 0.059(-0.020, 0.138) | 0.272(0.049, 0.495)* |
| Alcohol intake |  |  |  |  |  |  |
| Non-drinker (N,%) | 592 (17.62%) | Reference | Reference | Reference | Reference | Reference |
| 1-5 drinks/month (N,%) § | 1816 (54.06%) | 0.273(-0.403, 0.950) | -0.023(-0.052, 0.006) | 0.054(-0.069, 0.178) | -0.016(-0.100, 0.068) | 0.356 (0.135, 0.577)* |
| 5-10 drinks/month (N,%) | 331 (9.85%) | 0.342(-0.378, 1.063) | -0.015(-0.057, 0.028) | -0.119(-0.245, 0.006) | 0.188 (0.066, 0.310)* | 0.342(0.108, 0.576)* |
| 10+ drinks/month (N,%) | 620 (18.46%) | 0.492(-0.119, 1.102) | -0.012(-0.037, 0.013) | 0.072(-0.025, 0.170) | 0.009(-0.075, 0.092) | 0.361(0.149, 0.573)* |
| Smoking status |  |  |  |  |  |  |
| Never smoker (N,%) | 1643 (48.91%) | Reference | Reference | Reference | Reference | Reference |
| Former smoker (N,%) | 951 (28.31%) | -0.251(-0.667, 0.165) | -0.013(-0.033, 0.007) | 0.146(0.074, 0.218)** | -0.201(-0.284, -0.118)** | -0.037(-0.252, 0.179) |
| Current smoker (N,%) | 765 (22.77%) | 0.853(0.361, 1.345)* | -0.020(-0.040, 0.001) | 0.130(0.035, 0.224)* | -0.011(-0.084, 0.063) | 0.570 (0.330, 0.810)** |

† Early Adulthood: ages 20-44, Middle Age: ages 45-64, and Late Life: ages ≥65 ‡ Underweight (BMI values < 18.5); Normal weight (BMI values 18.5-24.9); Overweight (BMI values 25.0-29.9); Obese Class I (BMI values 30.0-34.9); Obese Class II (BMI values 35.0-39.9); and Obese Class III (BMI values≥ 40.0). § In this context, a “drink” refers to any of the following: a 12 oz. serving of beer, a 5 oz. glass of wine, or 1.5 oz. of liquor. **P-value <0.001, *P-value <0.05 WC:waist circumference; BMI: body mass index; SHBG: sex hormone-binding globulin; T/E_2_: testosterone/estradiol; FAI: free androgen index.

**Supplementary Table 3: Threshold effect analysis of waist circumference on sex steroid hormones using piecewise linear regression models**

| Exposure | Inflection point (cm) | Group | β (95% CI) P | P for log likelihood ratio test |
| --- | --- | --- | --- | --- |
| Testosterone | 97.6 | ≤97.6 | -0.190 (-0.212, -0.169) <0.001 | <0.001 |
|  |  | ＞97.6 | -0.079 (-0.092, -0.066) <0.001 |  |
| Estradiol | 104.5 | ≤104.5 | -0.000 (-0.001, 0.001) 0.592 | <0.001 |
|  |  | ＞104.5 | 0.004 (0.003, 0.005) <0.001 |  |
| SHBG | 99.1 | ≤99.1 | -0.036 (-0.039, -0.032) <0.001 | <0.001 |
|  |  | ＞99.1 | -0.004 (-0.007, -0.002) <0.001 |  |
| FAI | 98.6 | ≤98.6 | 0.013 (0.010, 0.016) <0.001 | <0.001 |
|  |  | ＞98.6 | -0.009 (-0.011, -0.007) <0.001 |  |
| T/E_2_ | 129.6 | ≤129.6 | -0.073 (-0.078, -0.069) <0.001 | 0.069 |
|  |  | ＞129.6 | -0.051 (-0.073, -0.030) <0.001 |  |

SHBG: sex hormone-binding globulin; T/E_2_: testosterone/estradiol; FAI: free androgen index

**Supplementary Table 4: Stratified Analysis of Waist Circumference and Sex Steroid Hormones**

| Covariate | N,% | Testosterone β(95%CI) *P* | *P* * | Estradiol β(95%CI) *P* | *P* * | SHBG β(95%CI) *P* | *P* * | FAI β(95%CI) *P* | *P* * | T/E_2_ β(95%CI) *P* | *P* * |
| --- | --- | --- | --- | --- | --- | --- | --- | --- | --- | --- | --- |
| Age (year) |  |  | 0.938 |  | 0.857 |  | 0.704 |  | 0.539 |  | 0.945 |
| Early Adulthood † | 1573 (46.83%) | -0.117(-0.141, -0.093) <0.001 |  | 0.002(0.001, 0.003) 0.001 |  | -0.015(-0.020, -0.010) <0.001 |  | -0.002(-0.005, 0.001) 0.088 |  | -0.075(-0.086, -0.064) <0.001 |  |
| Middle Age | 1132 (33.70%) | -0.120(-0.140, -0.099) <0.001 |  | 0.002(0.000, 0.004) 0.031 |  | -0.013(-0.018, -0.008) <0.001 |  | -0.005(-0.010, -0.001)0.028 |  | -0.073(-0.084, -0.062) <0.001 |  |
| Late Life | 654 (19.47%) | -0.110(-0.153, -0.067) <0.001 |  | 0.002(-0.001, 0.005)0.110 |  | -0.012(-0.018, -0.007) 0.005 |  | -0.004 (-0.011, 0.002)0.142 |  | -0.067(-0.084, -0.051) <0.001 |  |
| Race |  |  | <0.001 |  | <0.001 |  | 0.202 |  | 0.273 |  | 0.046 |
| Mexican American(N,%) | 543 (16.17%) | -0.098 (-0.128, -0.067) 0.018 |  | 0.002 (0.000, 0.004) 0.018 |  | -0.011 (-0.016, -0.006) 0.018 |  | -0.003 (-0.005, -0.001) 0.018 |  | -0.063(-0.072, -0.054) 0.018 |  |
| Other Hispanic (N,%) | 345 (10.27%) | -0.131 (-0.172, -0.090) <0.001 |  | 0.002 (-0.001, 0.004) 0.121 |  | -0.017 (-0.023, -0.011) 0.003 |  | -0.003 (-0.010, 0.004) 0.289 |  | -0.076(-0.096, -0.056) <0.001 |  |
| Non-Hispanic White (N,%) | 1320 (39.30%) | -0.114(-0.130, -0.097) <0.001 |  | 0.003 (0.002, 0.004) <0.001 |  | -0.016 (-0.019, -0.013) <0.001 |  | -0.001(-0.004, 0.002) 0.576 |  | -0.078(-0.087, -0.069) <0.001 |  |
| Non-Hispanic Black (N,%) | 653 (19.44%) | -0.128(-0.146, -0.110) <0.001 |  | 0.001 (-0.001, 0.002) 0.412 |  | -0.015 (-0.018, -0.013) <0.001 |  | -0.003(-0.006, 0.000) 0.072 |  | -0.061(-0.073, -0.048) <0.001 |  |
| Non-Hispanic Asian (N,%) | 381 (11.34%) | -0.149(-0.205, -0.093) <0.001 |  | 0.001 (-0.002, 0.004) 0.368 |  | -0.023 (-0.033, -0.014) 0.001 |  | 0.002(-0.009, 0.012) 0.646 |  | -0.083(-0.102, -0.065) <0.001 |  |
| Other Race (N,%) | 117 (3.48%) | -0.146(-0.187, -0.105) 0.006 |  | 0.000 (-0.004, 0.005) 0.738 |  | -0.017 (-0.038, 0.004) 0.068 |  | -0.004(-0.020, 0.012) 0.389 |  | -0.071(-0.103, -0.040) 0.002 |  |
| BMI(kg/m^2^) |  |  | 0.021 |  | <0.001 |  | <0.001 |  | 0.032 |  | 0.559 |
| Normal weight‡ | 885 (26.35%) | -0.112(-0.193, -0.030)0.013 |  | 0.001(-0.003, 0.005)0.559 |  | -0.024(-0.038, -0.010) 0.005 |  | 0.047(-0.005, 0.099)0.055 |  | 0.026 (-0.105, 0.159)0.006 |  |
| Underweight | 38 (1.13%) | 0.351(0.098,0.604) 0.016 |  | 0.010(-0.008, 0.029) 0.618 |  | -0.013(-0.046, 0.021) 0.621 |  | 0.011(-0.001, 0.023) 0.055 |  | -0.057 (-0.092, -0.022)0.006 |  |
| Overweight | 1282 (38.17%) | -0.135(-0.191, -0.080) 0.001 |  | -0.000(-0.003, 0.003)0.994 |  | -0.017(-0.026, -0.008)0.003 |  | -0.003(-0.013, 0.007)0.451 |  | -0.066(-0.100, -0.031)0.003 |  |
| Obesity Class I | 717 (21.35%) | -0.078(-0.144, -0.013)0.021 |  | 0.001(-0.004, 0.007)0.502 |  | -0.008(-0.018, 0.002)0.084 |  | -0.004(-0.016, 0.008)0.417 |  | -0.047(-0.081, -0.013)0.012 |  |
| Obesity Class II | 269 (8.01%) | -0.062(-0.232, 0.108)0.214 |  | 0.000(-0.009, 0.010)0.868 |  | -0.007(-0.038, 0.024)0.410 |  | -0.002(-0.025, 0.021)0.790 |  | -0.031(-0.097, 0.035)0.137 |  |
| Obesity Class III | 168 (5.00%) | -0.011(-0.076, 0.055)0.082 |  | 0.005 (0.001, 0.009)0.016 |  | 0.005(-0.007, 0.016)0.548 |  | -0.008(-0.017, 0.001)0.276 |  | -0.043 (-0.076, - 0.009)0.016 |  |
| Education level |  |  | 0.048 |  | 0.017 |  | 0.041 |  | 0.122 |  | 0.737 |
| Less than 9th grade | 306 (9.11%) | -0.122(-0.158, -0.085) <0.001 |  | 0.001(-0.001, 0.003) 0.190 |  | -0.018(-0.024, -0.012) <0.001 |  | 0.000(-0.005, 0.005)0.960 |  | -0.073(-0.096, -0.050) <0.001 |  |
| 9-11th grade | 444 (13.22%) | -0.100(-0.135, -0.066) <0.001 |  | 0.003(0.002, 0.004) <0.001 |  | -0.008(-0.015, -0.002)0.033 |  | -0.006(-0.011, -0.001)0.016 |  | -0.069(-0.081, -0.057) <0.001 |  |
| High school graduate/ GED or equivalent | 796 (23.70%) | -0.136(-0.158, -0.114) <0.001 |  | 0.001(-0.000, 0.003)0.057 |  | -0.017(-0.022, -0.012) <0.001 |  | -0.003(-0.008, 0.002)0.203 |  | -0.075(-0.084, -0.067) <0.001 |  |
| Some college or AA degree | 937 (27.90%) | -0.120(-0.145, -0.095) <0.001 |  | 0.002(0.001, 0.003)0.004 |  | -0.016(-0.019, -0.013) <0.001 |  | -0.002(-0.004, 0.001)0.169 |  | -0.075(-0.085, -0.065) <0.001 |  |
| College graduate or above | 876 (26.08%) | -0.105(-0.129, -0.081) <0.001 |  | 0.003(0.002, 0.004) <0.001 |  | -0.016(-0.021, -0.011) <0.001 |  | 0.001(-0.004, 0.005)0.664 |  | -0.075(-0.087, -0.064) <0.001 |  |
| Diabetes |  |  | 0.006 |  | 0.252 |  | <0.001 |  | 0.003 |  | 0.148 |
| Yes | 579 (17.24%) | -0.105(-0.146, -0.064) <0.001 |  | 0.002(0.001, 0.004) 0.038 |  | -0.007(-0.013, -0.002) 0.032 |  | -0.009(-0.015, -0.004)0.005 |  | -0.070(-0.080, -0.059) <0.001 |  |
| No | 2780 (82.76%) | -0.121(-0.139, -0.104) <0.001 |  | 0.002(0.001, 0.003) 0.001 |  | -0.017(-0.021, -0.013) <0.001 |  | -0.000(-0.003, 0.002)0.706 |  | -0.075(-0.083, -0.068) <0.001 |  |
| Session of blood sample collection | | | 0.234 |  | 0.020 |  | 0.703 |  | 0.049 |  | 0.004 |
| Morning | 1601 (47.66%) | -0.125(-0.147, -0.103) <0.001 |  | 0.002(0.001, 0.003)0.009 |  | -0.015(-0.020, -0.011) <0.001 |  | -0.002(-0.005, 0.001)0.195 |  | -0.072(-0.079, -0.065) <0.001 |  |
| Afternoon | 1239 (36.89%) | -0.111(-0.131, -0.090) <0.001 |  | 0.002(0.001, 0.003)0.007 |  | -0.017(-0.021, -0.013) <0.001 |  | 0.001(-0.003, 0.004)0.741 |  | -0.070(-0.079, -0.062) <0.001 |  |
| Evening | 519 (15.45%) | -0.108(-0.139, -0.077) <0.001 |  | 0.004(0.003, 0.006)0.001 |  | -0.012(-0.019, -0.006)0.003 |  | -0.005(-0.009, -0.001)0.046 |  | -0.091 (-0.106, -0.077) <0.001 |  |
| Physical activity time (hour / week) | | | 0.443 |  | 0.066 |  | 0.989 |  | 0.376 |  | 0.548 |
| Non-activity | 1907 (56.77%) | -0.120(-0.135, -0.105) <0.001 |  | 0.003(0.001, 0.004) 0.001 |  | -0.015(-0.019, -0.012) <0.001 |  | -0.002(-0.005, 0.001)0.124 |  | -0.078(-0.087, -0.069) <0.001 |  |
| 0.1-0.9 | 102 (3.04%) | -0.110(-0.461, 0.242) 0.058 |  | 0.002(-0.022, 0.026) 0.389 |  | -0.019(-0.077, 0.039)0.052 |  | 0.002(-0.037, 0.040)0.612 |  | -0.073(-0.186, 0.039)0.014 |  |
| 1.0-3.4 | 297 (8.84%) | -0.085 (-0.133, -0.036) 0.004 |  | 0.004(0.002, 0.006) 0.003 |  | -0.014(-0.022, -0.006) 0.009 |  | 0.002(-0.007, 0.011) 0.631 |  | -0.076(-0.092, -0.060) <0.001 |  |
| 3.5-5.9 | 125 (3.72%) | -0.104(-0.162, -0.046) 0.016 |  | 0.002(-0.001, 0.004)0.061 |  | -0.017(-0.028, -0.006)0.016 |  | 0.002(-0.008, 0.011)0.068 |  | -0.068(-0.091, -0.045)0.016 |  |
| ≥6 | 928 (27.63%) | -0.123(-0.142, -0.104) <0.001 |  | 0.001(0.000, 0.003)0.034 |  | -0.016(-0.019, -0.013) <0.001 |  | -0.002(-0.004, 0.001) 0.175 |  | -0.070(-0.081, -0.059) <0.001 |  |
| Alcohol intake |  |  | 0.332 |  | 0.004 |  | 0.330 |  | 0.900 |  | 0.304 |
| Non-drinker | 592 (17.62%) | -0.131(-0.161, -0.102) <0.001 |  | 0.001(-0.000, 0.002)0.228 |  | -0.018(-0.023, - 0.012) <0.001 |  | -0.002(-0.005, 0.002)0.355 |  | -0.068(-0.080, -0.056) <0.001 |  |
| 1-5 drinks/ month § | 1816 (54.06%) | -0.119(-0.140, -0.098) <0.001 |  | 0.002(0.001, 0.003)0.001 |  | -0.017(-0.022, -0.012) <0.001 |  | -0.000(-0.004, 0.004)0.900 |  | -0.076(-0.084, -0.068) <0.001 |  |
| 5-10 drinks/ month | 331 (9.85%) | -0.103(-0.140, -0.067) <0.001 |  | 0.004(0.002, 0.005) 0.001 |  | -0.012(-0.019, -0.005)0.004 |  | -0.004(-0.011, 0.003)0.208 |  | -0.079(-0.094, -0.063) <0.001 |  |
| 10+ drinks/ month | 620 (18.46%) | -0.106(-0.133, -0.078) <0.001 |  | 0.003(0.001, 0.005)0.014 |  | -0.011(-0.016, -0.007) <0.001 |  | -0.004(-0.009, 0.001)0.121 |  | -0.072(-0.082, -0.061) <0.001 |  |
| Smoking status |  |  | 0.516 |  | 0.360 |  | 0.629 |  | 0.315 |  | 0.010 |
| Never smoker | 1643 (48.91%) | -0.116(-0.136, -0.096) <0.001 |  | 0.002(0.001, 0.003) <0.001 |  | -0.016(-0.021, -0.012) <0.001 |  | -0.000(-0.003, 0.003) 0.770 |  | -0.073(-0.080, -0.067) <0.001 |  |
| Former smoker | 951 (28.31%) | -0.128(-0.161, -0.095) <0.001 |  | 0.003(0.001, 0.004) 0.007 |  | -0.014(-0.019, -0.010) <0.001 |  | -0.005(-0.010, 0.001) 0.078 |  | -0.085(-0.099, -0.072) <0.001 |  |
| Current smoker | 765 (22.77%) | -0.106(-0.131, -0.081) <0.001 |  | 0.002(0.000, 0.004)0.031 |  | -0.013(-0.020, -0.007) 0.002 |  | -0.001(-0.007, 0.004) 0.632 |  | -0.066(-0.078, -0.054) <0.001 |  |

*P* * *P* for interaction † Early Adulthood: ages 20-44, Middle Age: ages 45-64, and Late Life: ages ≥65 ‡ Underweight (BMI values < 18.5); Normal weight (BMI values 18.5-24.9); Overweight (BMI values 25.0-29.9); Obese Class I (BMI values 30.0-34.9); Obese Class II (BMI values 35.0-39.9); and Obese Class III (BMI values ≥ 40.0). § In this context, a "drink" refers to any of the following: a 12 oz. serving of beer, a 5 oz. glass of wine, or 1.5 oz. of liquor. WC:waist circumference; BMI: body mass index; SHBG: sex hormone-binding globulin; T/E_2_: testosterone/estradiol; FAI: free androgen index. In the stratified analysis, all models were fully adjusted for variables including age, race, education, ratio of family income to poverty, diabetes, blood collection session, cotinine, physical activity, alcohol and smoking, excluding the subgroup variable.
